# Recognizing, normalizing and articulating: An approach to highlight plural values of water ecosystem services in Colombia

**DOI:** 10.1016/j.heliyon.2022.e10622

**Published:** 2022-09-17

**Authors:** Andres Suarez, Cesar Augusto Ruiz-Agudelo, Paola Arias-Arévalo, Gloria Y. Flórez-Yepes, Nicolas Arciniegas, Luis A. Vargas-Marín, Alejandro Marulanda, Jesica Ramirez, Edisson Castro-Escobar, Juan C. Bastidas, David Blanco

**Affiliations:** a Department of Civil and Environmental, Universidad de La Costa, Calle 58#55-66, Barranquilla 080001, Colombia; b Doctoral Program in Environmental Sciences and Sustainability, “Jorge Tadeo Lozano” University, Bogotá 111311, Colombia; c Department of Economics. Faculty of Social and Economic Sciences, University of el Valle, Ciudad Universitaria Meléndez. Cali, Valle del Cauca 760032, Colombia; d GIDTA Research Group, Catholic University of Manizales, Manizales 170001, Colombia; e Wildlife Conservation Society -WCS, Colombia Program, Cali 760046, Colombia; f Environment and Development Research Center (CIMAD), University of Manizales, Carrera 9a #19-03 B/Campo Hermoso, Manizales 170001, Colombia; g Water Resources, Mg. of Engineering. Unal, Universidad Nacional -UNAL, Manizales 170001, Colombia; h Research Group in Natural Resources and Environment (GIRNMAC), Regional Autonomous Corporation of Caldas –CORPOCALDAS, Manizales 170001 Colombia

**Keywords:** Nature contributions to people, Plural valuation, Social participation, Integration: decision making

## Abstract

The dialectical relationship between ecosystems and society is complex; therefore, holistic approaches are required to address this complexity. This view also stands out in the ecosystem services valuation field, where different scholars and global platforms have drawn attention to the need to incorporate plural valuation initiatives at decision-making. In this sense, through a comprehensive design, we conducted a multi-layered valuation of ecosystem services, and we highlighted multiple values in two areas of the province of Caldas, Colombia. We proposed a three-phase valuation process called *Recognizing*, *Normalizing* and *Articulating* values. Then, in cooperation with the regional environmental authority, we obtained different water-related ecosystem services values. Our results showed some warnings: first, we found mismatches between ecosystem services values; second, people assigned high values to ecosystems but the actual capacity of ecosystems to support ES is low. Finally, monetary values were marginal compared to social and ecological values. We conclude by saying that the more strata are assessed, the more values appear in the valuation scenarios, and those values could be conflicting. Our results have political implications, since they highlight the need to incorporate plural values as a fundamental tool for planning and land use in real scenarios where conflicts of interest and values are evident.

## Highlights

• Plural valuation of ecosystem services is a field in construction and we add methodological insights.
• We provided a multilayered-valuation of ecosystem services to elicit plural values related to water.
• We used a pragmatic philosophy following a multimethod research design with a mixed-approach.
• Water ecosystem services have different values and we proved in two areas from Colombia.
• The more layers unfolded, the more values found according to our approach.

## 1 Introduction

In the last years, a significant body of literature has called attention to the need to go beyond monetary, instrumental, or monistic approaches to value Ecosystem Services –ES (Jacobs et al., 2020; Rincón-Ruiz et al., 2019; Jacobs et al., 2016; Pascual et al., 2017; Villegas-Palacios et al., 2016). Furthermore, scholars have stressed the need of considering particular context-specific circumstances which influence valuation processes and values (Díaz et al., 2018; Pascual et al., 2017; Pandeya et al., 2016). In this line, Plural Valuation of ES has been defined as “a *political science process that assesses the multiple values attributed to nature by social participants…as well as how these values relate to each other, and how this information can guide decision-making. The multiple values are articulated through a context-specific process that takes into account worldviews, socio-ecological interactions, power relations, and the valuation process itself”* (Rincón-Ruiz et al., 2019:pp2)*.*

After several years of scientific debate among monetary and pluralistic valuation scholars (e.g. Gsottbauer et al., 2015; Kallis et al., 2013) “*the dust seems to be settling*” (Jacobs et al., 2016: pp214): the pluralistic valuation (PV) have gained a relevant academic terrain. Two processes have been crucial towards mainstreaming plural valuation in the academic and political field. One of them has been the participation of plural valuation from ecological economics and sustainability fields, in political science such as The Economics of Biodiversity and Ecosystem Services (http://teebweb.org/) and the Intergovernmental political science Platform on Biodiversity and Ecosystem Services -IPBES (https://ipbes.net/). The second one has been the consolidation of academic global networks that have advanced the plural valuation agenda through research projects, academic congresses, and high-impact publications. This global academic network has been called the new valuation school (Jacobs et al., 2016). This school has contributed to change a monistic monetary valuation worldview towards an holistic one (Pereira et al., 2020). Plural valuation may contribute towards the achievement more equitable and sustainable outcomes (Zafra-Calvo et al., 2020), for instance by supporting efforts to minimize negative social externalities (Phelan and Jacobs, 2016), or by finding ways of mitigating or preventing social conflicts associated to the multiple and often conflicting values among decision makers and other local participants (Rincón-Ruiz et al., 2019). Furthermore, PV may support adaptive management (Wam et al., 2016) and ecosystems sustainability (Herbst et al., 2020).

Despite PV conceptual contributions and its potential towards advancing sustainability and equity goals, the operationalization of value pluralism in ES assessments is still slight (Arias-Arévalo et al., 2018). Some of the challenges of plural valuation operationalization includes: (i) the complexity it involves (Jacobs et al., 2016), there are limitations and uncertainties in integrating ecological, economic, and social values of ES (Mueller et al., 2016), political will (Rincón-Ruiz et al., 2019), and the need to negotiate multiple adapt knowledge system and epistemologies in valuation process (Gómez-Baggethun and Martín-López, 2015; Spash, 2012).

Although bringing about plural values might be more expensive in terms of the resources and data needed, it explicitly addresses the gaps in knowledge, offering a way to articulate among different value domains (Maydana et al., 2020). Nevertheless, by examining six review studies of tools for plural valuation, Neuteleers and Hugé (2021) revealed there is little attention for value pluralism, no matter the existence of a political science consensus regarding the need to recognize this pluralism in the field. Thus, as pointed out by Zafra-Calvo et al. (2020), it is necessary to invest in efforts to integrate PV through action-oriented approaches. Considering the above and taking into account all the complexity of the PV of ES, given that the valuation of ES remains challenging, especially at a local scale and in data scarce regions (Pandeya et al., 2016). In this line, as Brück et al. (2022) defined, there is a need to perform disaggregated analysis considering who benefits from which particular values, where and when.

Some empirical evidence has approached comprehensively to PV and has contributed to mainstreaming this new valuation school. What is clear in those studies is that bringing out PV call for the integration of several approaches and multiple perspectives. For instance, Kenter (2016) comprehensively got and understood cultural ES by integrating deliberative monetary valuation, subjective well-being and psychometric approaches. In addition, by using multi-criteria analysis, Liquete et al. (2016), and Saarikoski et al. (2016), assessed and integrated multiple values to inform decision-making. Further research has included mixed-methods to assess since a plural perspective, how the lack of value integration aggravates social externalities (Phelan and Jacobs, 2016). Likewise, multiple methods have been applied to understand how plural values inform planning-scenarios (Friedrichsen et al., 2021; Rojas et al., 2019; Peh et al., 2016), and contribute to the land use planning, through a set of comprehensive indicators (Maydana et al., 2020). Further PV studies have focused strongly on social perceptions by considering context-specific characteristics and broad value domains such as instrumental, intrinsic and relational (Coelho-Junior et al., 2021; Gale and Ednie, 2020; Arias-Arévalo et al., 2017).

Therefore, we found all those studies meaningful to guide PV studies and we aim to add concerns and warnings to mainstream plural valuation of ES by considering a study-case application. However, through our research we fill gaps identified in literature related to the inclusion of more participatory approaches in PV(Zafra-Calvo et al., 2020), and particularly PV in Latin-American context and in Colombia as well. Likewise, this contribution aims to discuss the applicability of PV on scenarios of socio-environmental planning and ecosystems management, in a specific case in the Colombian Andean region where there is a lack of inclusion of PV in decision making. In this line, all literature on PV has been meaningful for the academic and decision-making process, and we acknowledge all contributions to the integration of PV in ES research. Therefore, our focus is related to answering how to integrate the operationalization of the PV in a concrete case for decision-making in Latin-America, particularly in Colombia. This means that we deepen into the valuation of one dimension of nature *in particular*, which is the driver of management and politics in the study area, namely water ecosystem services (Water regulation and supply). In addition, we intended to evidence the following assumptions: *the same ecosystem service is valued differently taking into account different approaches and scales*, and *the results of a plural valuation comprehensively inform the decision-making process*.

### 1.1 Study area

**Figure 1:**
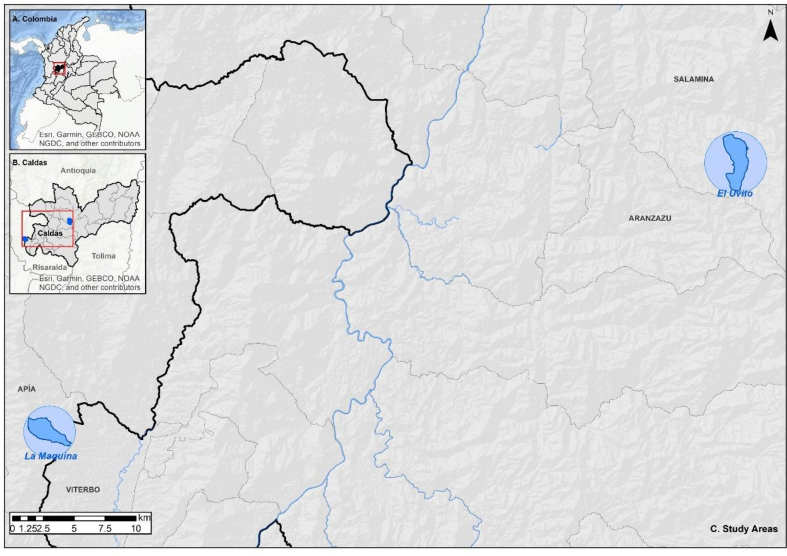
Study area.

To select our study area and for applying a PV, we selected two case studies meaningful for political interventions. Therefore, we looked at in our study areas which represent environmental restrictions for productive land uses, and they represent a strategy for the conservation of water resources at the regional level (Figure 1). These zones are the supplying areas of urban collective aqueducts, which are a special category for water conservation and planning in the region.

Supplying area El Uvito: This area located in the municipality of Salamina in the province of Caldas (South west Colombia), which has a population of 8,841 inhabitants. El Uvito reach an area of 564.2 ha, which rises at an elevation of 3,000 m above sea level and flows along 5.75 km into the Pocito River at an elevation of 2,000 m above sea level (UTP, 2015). The annual precipitation average is 1,842 mm and average temperature is 11 °C. Along the riverbed, there is natural forest and bushlands. The predominant vegetation cover is clean and weeded pasture corresponding to 45% of the total area, followed by heterogeneous agricultural areas (28%) mainly coffee and avocado, and finally 26% is forest. Although El Uvito currently faces middle levels of water supply-shortages, it is projected to be the first alternative for water supply for the municipality of Salamina, which makes it a relevant local and regional area for watershed conservation. however, nowadays, El Uvito supplies water to more than 3000 dwellers.

On the other hand, the supplying area *La Máquina*, located at 1,021 m above sea level, *La Máquina* is essential for the provision of water service for the aqueduct of the municipality of Viterbo (Province of Caldas), a municipality with 3,446 dwellers. The annual average of precipitation is 2,553 mm and the average temperature is 17 °C nowadays, la Máquina supplies with water around 500 rural and urban dwellers. La Máquina is a 392.6 ha area, mainly dominated by agricultural activities (94%), and followed by forests (4%) and pastures (2%). Only 7% of the area belongs to Viterbo, the remaining 93% belongs to other municipalities (UTP et al., 2015). The presence of coffee and banana crops, as well as livestock and the presence of dispersed rural housing, threatens water quality with the possible delivery of domestic and non-domestic wastewater, overgrazing, and contamination of the water resource with pesticides and chemical fertilizers required for these crops., La Máquina currently faces a very high level of risk of water supply shortages.

We call the attention to the environmental authority's narrative regarding the importance of water supplying areas for urban inhabitants. According to Corpocaldas (Regional Environmental Authority), the main objective of a supplying area is to get the provision of the ecosystem service of water supply for human consumption, through the protection and/or sustainable use of existing land covers, contributing to water regulation and pollution reduction. Therefore, the environmental authority has developed rules regarding to the type of land uses allowed in these areas. On the one side, the main land use relates to watershed conservation; compatible uses are agro-forestry and some conditional uses are related to public use. In addition, there is a long list of forbidden and conditioned uses. Therefore, as starting point, the supplying areas aim to strictly protect and conserve water, and the valuation language for these areas are mainly supported by an ecological perspective which guide land use planning.

## 2 Methodology

We proposed a multi-layered ES valuation, which we defined as the integrated analysis of the emergent ES water-related values. These values consist in different types (i.e. ecological, social, monetary), and they are articulated at different scales. To address this, we followed a Pragmatic philosophy (Melnikovas, 2018; Peck and Khirfan, 2021); moreover, we followed the call for applying multiple methods aimed at obtaining ES plural values (Jacobs et al., 2016). Therefore, we combined several methods, and we followed a mixed quantitative-driven methodology, following an exploratory sequential design in which qualitative data was acquired first, followed by quantitative data, and finally a process of interpretation of results (Creswell and Creswell, 2018). In addition, following Jacobs et al. (2016) suggestion of promoting cooperation among scientists, decision-makers, practitioners and policymakers, this research was funded between the University of Manizales and the environmental authority of Caldas-Corpocaldas, and the research process included Corpocaldas in the design, development and final discussion of the research.

The particular role of Corpocaldas was threefold: firstly, supporting the selection of the study area, secondly, in the process of methodological validations and, finally, complementing the results to be included in subsequent political interventions. In that sense, after discussions about the accuracy for applying the research, Corpocaldas provided three criteria for selecting the areas: (i) areas with risk of water shortage, (ii) affectation degree due to land cover change, and (iii) pressures generated by productive activities. Therefore, in this conjoint discussion, we selected two supplying areas as case studies.

### 2.1 Research phases

We followed three general phases that we called Recognizing – Normalizing - Articulating, and in each of them, different steps and methods to deploy the valuation layers. Although TEEB (2010) proposed a three-step valuation process called Recognizing – Demonstrating - Capturing, we took distance from this approach in at least two aspects: first, we were not interested in demonstrating only monetary values of ES, in fact we assumed them to be marginal and contextual (Arias-Arévalo et al., 2018; Kallis and Bagetun, 2013). For this reason, we needed a value normalization process. Second, we were not interested in providing an “*introduction of mechanisms that incorporate ecosystem values into decision making, through incentives and price signals*” (TEEB, 2010: pp12). Rather, we opted to articulate values in all their different dimensions at decision making through a dialectical process for political interventions.

Therefore, the initial phase was to *Recognize* different ES values. This means that social values, ecological values and monetary values were obtained. Then, as the values differ with each other, we carried out a *Normalization* of these values. Finally, we provided an *Articulation* of the recognized values through a dialectical narrative where all of them were highlighted.

#### 2.1.1 Phase 1: recognizing values

**Figure 2:**
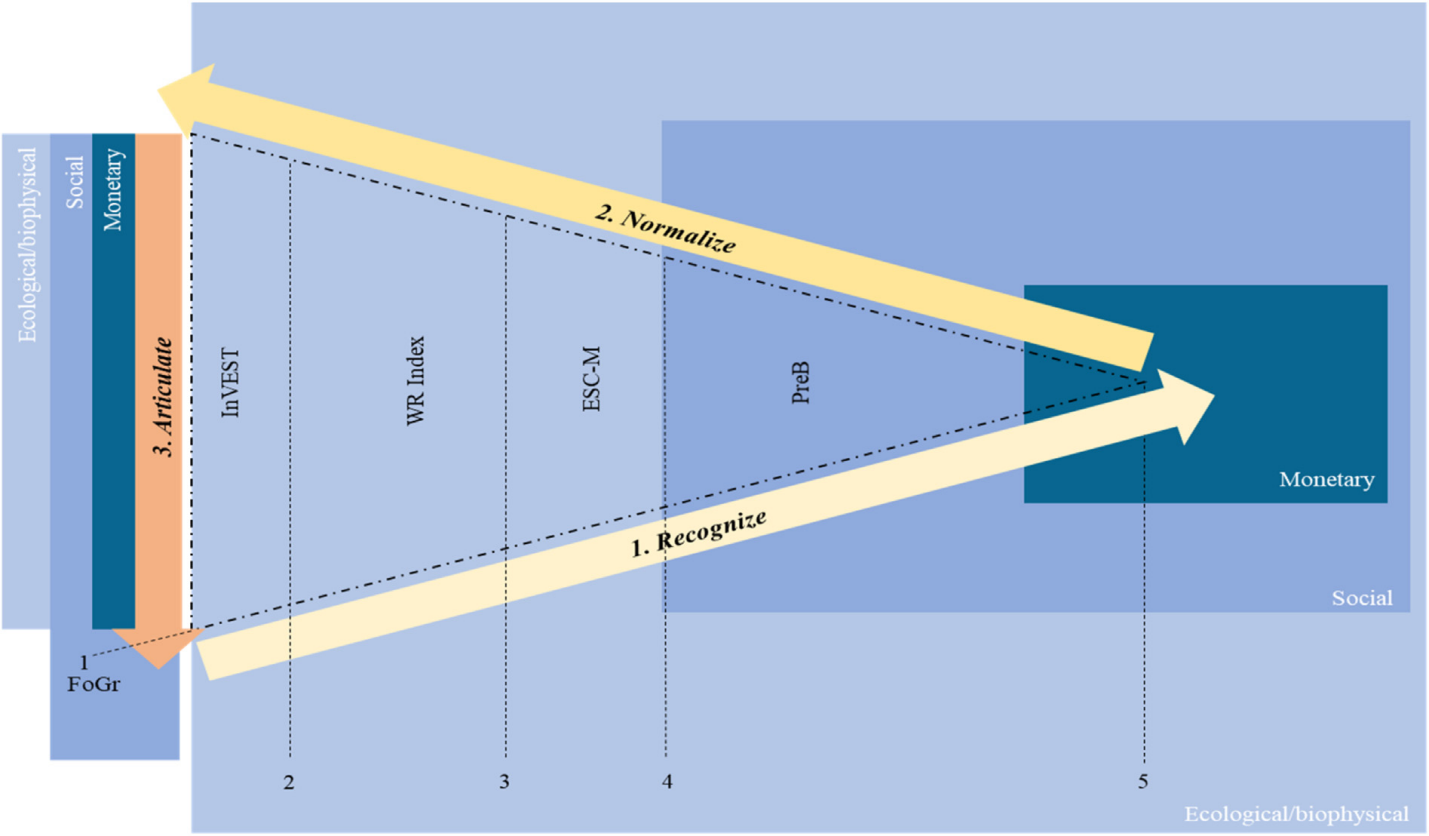
Multi-layered valuation. Each box represents value dimensions embedded in value types. The widest value type involves ecological values, the next type represents social values and finally, the smallest type shows monetary values. FoGr: focus groups; WR Index: water regulation Index; ESC-M: ecosystem services capacity matrix; PreB: a preference-based questionnaire.

As a starting point, we developed focus groups in both municipalities in order to capture the problematic context related to the supply areas; also, this lets us set the multi-layered valuation around the ES's more significant for the involved participants (Figure 2, transversal line). No ethical approvals were required for the study, but the consent of participants. Overall, after the problem identification (1), we proposed four additional methods to address the first phase related to *Recognize* values (Figure 2). As different valuation methods capture different values (Dendoncker et al., 2018) and have different suitability to obtain them (Jacobs et al., 2018), we proposed the following methods: (2) application of the InVEST tool related to water regulation; (3) description of the water regulation index; (4) generation of an ES capacity matrix and (5) the recognition of preference-based willingness to pay and social perceptions on ES. All the methods were related to water ES for two reasons: first, the supplying areas aim to protect water resources, and second, as a result of the focus groups*,* the involved participants prioritized water ecosystem services.

##### 2.1.1.1 Focus group

Focus groups provide a way to gather information from a small group, with the facilitation of the researchers (Biggs et al., 2021). Then, we developed focus groups aimed at acknowledging the problems, conflicts, as well as the potentialities of the area. The participants in groups identified the vision they had of the area, and finally, the participants provided a general identification of the benefits they perceived from supply areas. We asked two main questions for the groups: what are the most significant problems in the area? In addition, what are the most important benefits for them, provided by the supply area? Through these focus groups, we wanted to make echo of the voice of the participants. Therefore, following Muñoz-Rios et al. (2020) suggestion, to consider the qualitative results of the participants and given the relativism of the language, we combined some original sentences in Spanish with a short interpretation through “square-shaped” parentheses ‘[]’.

#### 2.1.2 Application of the InVEST tool related to water yield

We performed the estimation of supply area water yield in 2019 using spatial modeling, based on the InVEST 3.9.0 “Water yield” tool. This is based on the Budyko curve of hydrothermal balance and average annual precipitation (Zhang et al., 2004), and it is adapted to be calculated using raster inputs. We obtained climatic and biophysical data from different national and global sources (Supplementary Material A1, Table S1). Later on, we standardized to cell size and extent of 100 m to manage a scale according to the study area, and thus facilitate the analysis per pixel, since the output of this model provides us with the annual water yield for each cell and the geographic distribution pattern. We used the output to calculate quantiles to classify the raster into high medium and low yield to facilitate the later process of value normalization.

#### 2.1.3 Description of the water regulation index

Downscaling in our approach, the water regulation displays the capacity to keep water and defines the water regime in a sub-basin for a catchment point (Table 1). We obtained flows through hydrological modeling, performing the water balance that converts physical and climatic conditions into flows on a daily scale considering 35 years of analysis. Once we obtained the flows from highest to lowest and connected to the Weisbull probability function, we saw the Flow Duration Curve from we examined the water regulation Index from the area under the curve of the average over the area under the total curve. The water regulation Index is an indicator used to determine the subzones in conditions of higher or lesser capacity to retain and regulate water (IDEAM, 2013). It is calculated following Eq. (1):

(1) $$WRI=\frac{Vp}{Vt}$$

Where:

**Table 1:** Categories for the index of water retention and regulation.

WRI values	Category	Characteristics
*> 0,85*	Very high	Very high capacity of the basin to retain and regulate water
0.75–0.85	High	High capacity of the basin to retain and regulate water
0.65–0.75	Middle	Middle capacity of the basin to retain and regulate water
0.50–0.65	Low	Low capacity of the basin to retain and regulate water
<0.50	Very low	Very low capacity of the basin to retain and regulate water

Source: IDEAM (2013).

*WRI:* Water retention and regulation index.

*Vp:* Volume represented by the area below the average flow line on the daily flow duration curve.

*Vt:* Total volume represented by the area under the daily flow duration curve.

The results are categorized in the next form:

In addition, we considered a hypothetical historical scenario regarding a 100% forest cover and only one Ha of forest cover in both supplying areas to test if the water regulation index changed. We used the information provided in here to define the levels and attributes in the choice experiment model.

##### 2.1.3.1 Generation of the ecosystem services capacity matrix

For finding the capacity of ecosystems to generate ES, we adapted the method proposed by Campagne and Roche (2018). This is composed by filling out a matrix (rankings) that crosses forest cover vs. ES. Therefore, experts and knowledgeable people on issues related to biodiversity, ecosystems and ecosystem services carried out this qualification. The rating assigns a scale from zero to five, assuming the following scale of capacity to generate ecosystem services: not relevant (0); low capacity (1); relevant capacity (2); medium capacity (3); high capacity (4), and very high capacity (5). To decide if there was reliability among the ratings provided by the experts, we applied Krippendorff's alpha (inter-reliability index) (Campagne and Roche, 2018; Gwet, 2015). This alpha states the least acceptable value of reliability for a measurement instrument by different experts is *α= 0.667*. For this, we used SPSS® statistical package.

##### 2.1.3.2 Recognition of preference-based willingness to pay and social perceptions

We provided a pilot survey to 30 people in both municipalities in order to calibrate the instrument. As we asked for Likert scale, we performed a reliability analysis using the Cronbach's; we achieved an acceptable reliability level (α *= 0.70*). Following Raymond and Kenter (2016) observations on social values, through this method we identified individual opinions of worth about ES and we included expressions of preferences in terms of metrics. Therefore, for the social perspective analysis, we asked for socio-demographic information. In addition, we asked for the living time in the area, and perceptions about the current state of the supply area. Moreover, we identified the area's contributions to water ES. Finally, we asked for ranking the importance assigned to the area for maintaining water (perceived capacity).

To obtain the willingness to pay (WTP), we employed choice experiments (Koemle and Yu, 2020). Choice experiments allow valuing the changes in attributes that determine the supply of ES by presenting alternatives to a scenario called *status quo* and based on this, different values to pay. We constructed the valuation scenarios considering inputs from steps 1, 2 and 3 (Table 2). After a pilot survey with an open-ended WTP question (n = 30), we performed and adjusted surveys to obtain WTP in both municipalities through the application of a simple random sampling in October 2021 in both areas.

**Table 2:** Choice sets applied in the study cases.

Attribute	Levels	Attribute	Levels
Forest cover	Increasing	Hydrologic regulation	Increasing
(Total area of forest in the area)	Maintaining	(Capacity to maintain water supply)	Maintaining (SQ)
	Decreasing (SQ)		Decreasing
Attribute	Levels	Attribute	Levels
Productive activities	Increasing (SQ)∗	Willingness to pay	$ 0 (SQ)
(Agriculture and livestock)	Maintaining (SQ)∗	(preferences for paying or not)	$3.000
	Forbidding		$5.000
			$10.000

SQ = Status Quo. ∗ In Viterbo village, the SQ relates to an increasing in productive activities, in Salamina village the scenario is steady.

**Figure 3:**
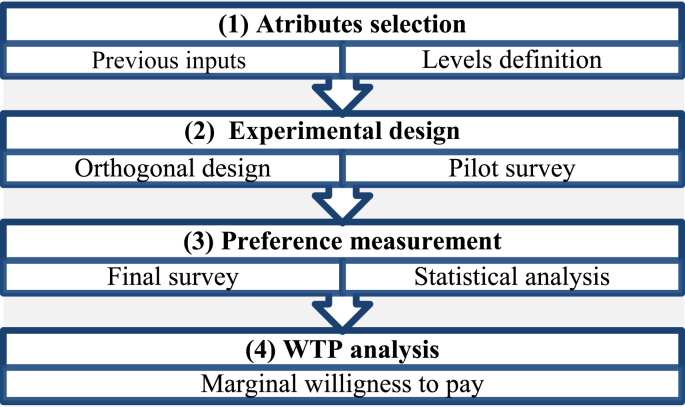
Choice experiment approach.

We considered a confidence level of 95% and an error of 10%. We developed face-to-face surveys, and we distributed the sampling process in both urban areas in a random fashion. In this sense, we addressed the method through the process depicted in Figure 3. To see detailed description of the valuation scenario, the questions, the attributes and levels, and general results, please see Supplementary Material A2, Table S2, S3 and S4, and Supplementary Material B.

To interpret the information, we performed descriptive and non-parametric statistics to find differences between social perspectives in both municipalities. To that end, we applied a Mann-Whitney U-Test (*p < 0.05*). To determine the WTP, we applied a mixed effect logit. We used Stata 14® statistical package.

#### 2.1.4 Phase 2: value normalization

For this analysis, we normalized one social value (*social perception about the supplying area's capacity to provide water*), ecological values from the matrix (*water provision capacity, water regulating capacity*), ecological value from InVEST (*water yield*), ecological value from the water regulation index, and finally the monetary value. Normalization has been used to minimize complexity in understanding the values (Wam et al., 2016), and to put in context the values elicited (Lau, 2015). We mention that we proceed with a particular step with InVEST results and monetary values. For instance, in the perception-based values, we obtained Likert scales, as well as in the ES capacity matrix. Therefore, minimum values were one, and maximum values were five. The water regulation index is in fact a 0–1 scale; however, the InVEST and results of WTP are not. Therefore, to normalize the InVEST values we identified the minimum and the maximum level of water yield provided in our analysis, and we proceeded to normalize. On the other hand, to normalize monetary values, we selected the international Dollar (Int$), which represents the value of the US dollar in terms of purchasing power. Then, we converted Colombian pesos (COP$) to Int$ using purchasing power parity-adjusted exchange rates (de Groot et al., 2020). For Colombia, in 2020 this value was one Int$ = 1,352 COP$. Therefore, the minimum value was zero Int$ corresponding to WTP = 0, and the maximum value was related with ES values reported by de Groot et al. (2020), framing our area into the tropical forest ecosystems category (Int$/hectare/year), and considering water provision and water regulation ES.

#### 2.1.5 Phase 3: articulation process

Following the insights provided by Villegas-Palacio et al. (2016) regarding PV initiatives, it is necessary to include political will from policy makers to implement management plans according to results given by the studies (Villegas-Palacio et al., 2016). In this sense, we constructed conjointly with the environmental authority a dialectical narrative in which we combined the obtained values to provide context and nuances among values. Furthermore, considering all values, we proposed guidelines to articulate the values in the decision-making process.

## 3 Results

### 3.1 Value recognition

#### 3.1.1 The focus groups

Overall, we worked with 16 people in both municipalities (eight *per* supply area). These people were part of community action boards, which had some relationship with the interest area, and they were related to the supply areas in different ways.

##### 3.1.1.1 El Uvito (salamina village)

In response to the guiding questions, the environmental problems identified in the supply areas by the participants were the expansion of the agricultural frontier with avocado production promoted by multinationals:

“los aguacateros son los más contaminantes de las aguas [*avocado growers are the most water polluters*]. Siempre se habla en las reuniones que se están apoderando de los recursos hídricos a través de las concesiones [*they are taking possession of the water through public permission]”*

“Estamos viendo la destrucción aterradora de los bosques [*we are facing a huge forest destruction*]…. por culpa de estos aguacateros que vinieron de otro país donde ya acabaron con su medio ambiente [*those foreign avocado growers destroyed the environment in their country*]”

“vamos a tener un problema muy grande con Salamina porque los aguacateros están acabando las reservas hídricas [*we are going to face big problems, because they -avocado growers- are deppleting water reserves*]”

In addition, the participants mentioned the need to increase the forests for higher water regulation, because of the historical decreasing in water flows over time.

“El área debería ser comprada para realizar un proceso de reforestación para cuidar el agua [*the area should be bought for water conservation*]”.

“se debe aumentar más el bosque para que el agua venga más fortalecida, tratar de que no se agote [*the forest areas should be increased to improve water provision*]”.

Regarding the potential of the area, the community highlights the scenic beauty and the natural landscape, which has been used for tourism activities and bird watching, as well as the human quality of the people as one of the greatest assets of the area. Finally, the participants prioritized the ES that in their opinion are the most important in El Uvito: water supply, air purification, and scenic beauty.

##### 3.1.1.2 La Máquina (viterbo village)

The participants highlighted the positive public management that has been done around the protection of water heritage, expanding the vegetation cover by the environmental authorities. In addition, they pointed out scenic beauty and the potential for ecotourism. Regarding the ES mentioned by them, the participants highlighted water regulation and the fact that the area promotes habitat conservation and species richness. On the other hand, the main environmental problems identified were the threat to the water source because of its neighboring municipality has a low interest in conserving their forest areas (border conflict due to supply area's shared jurisdiction):

“los de Risaralda no nos ayudan a cuidar, no hay control en la frontera agrícola [*people from Risaralda* (adjacent municipality) *do not help us, they do not control their agricultural frontier*]”

However, even in Viterbo there is a concern regarding the increase of the agricultural frontier, mainly for the avocado growing, and the fact that the area is attractive for recreational houses.

“el municipio ha sufrido un crecimiento demográfico y la gente está buscando este municipio para vivir aquí…. Los dueños de predios ven oportunidad de negocio y están vendiendo en áreas donde se puede afectar la provisión de agua [*the municipality has suffered a demographic growth and people are looking for this municipality to live here* .... *Property owners see business opportunities and are selling in areas where water supply can be affected*].”

Considering the needs and the discussions in the focus groups, we aimed our multi-layered valuation towards the recognition of the plural values related to the supply areas, but mainly by highlighting the importance of these areas for supplying water.

#### 3.1.2 The InVEST model

**Figure 4:**
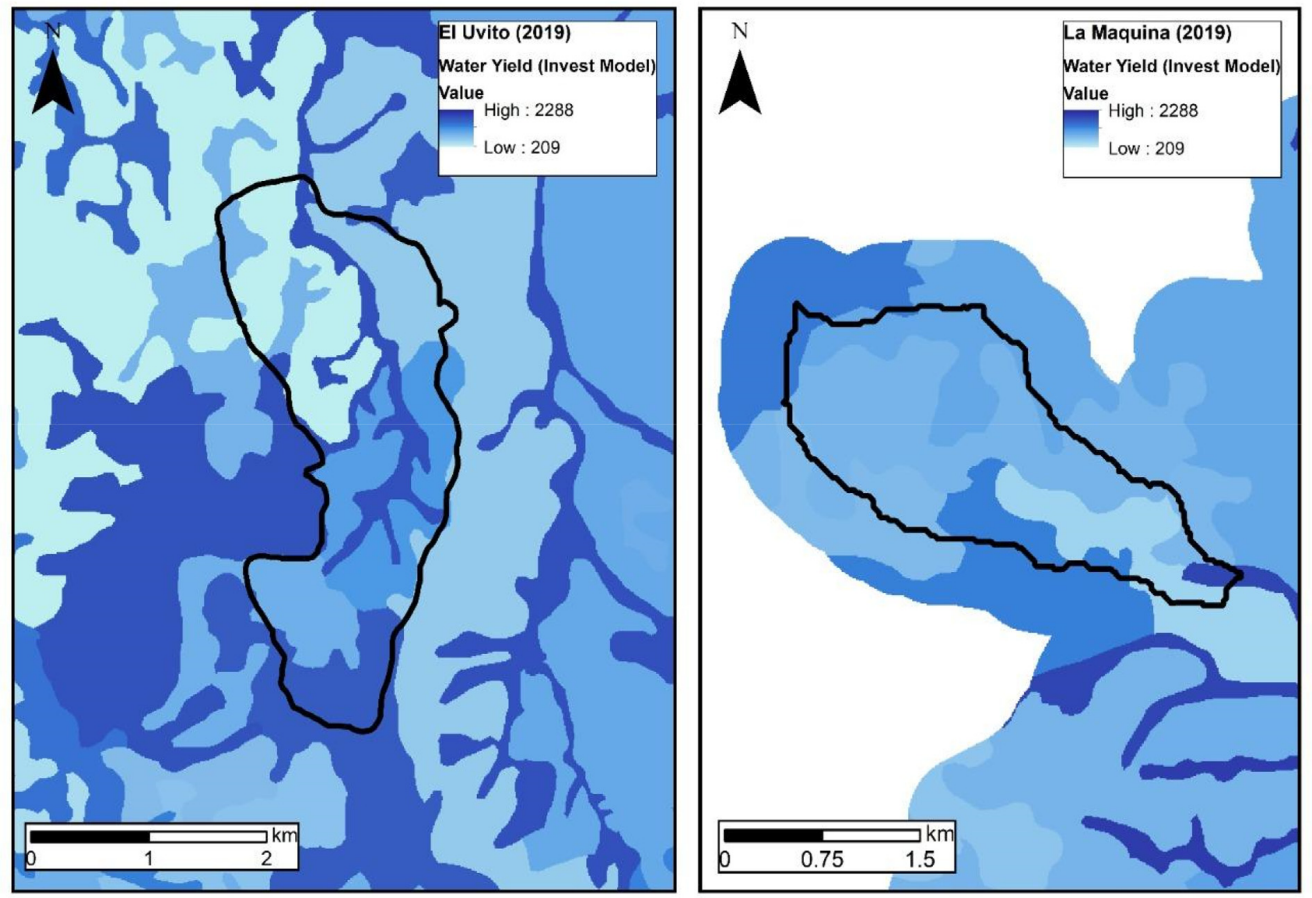
InVEST water yield modeling output. The values are in mm/year.

The InVEST output indicates that in El Uvito an average water yield of 1,083.2 mm was found, and in La Máquina 1,030.5 mm (Figure 4). The water production for the two supply areas had a quantitatively heterogeneous distribution, marked by the land cover (Supplementary material A3, Figures S1 and S2). This indicates that for these areas of water importance, the uptake of the resource is highly influenced by the type of land use found in the landscape, since speaking in terms of water conservation per cell, the high values were mainly concentrated in areas of native forests and shrublands. The medium yield area was located in areas of crops or productive vegetation, and finally, the low values lie in areas of pastures and bare soil.

The trend points to the importance of having natural vegetation, to maintain and conserve water in the strategic points of water source and distribution in the communities since they reduce surface runoff and effectively intercept the water obtained by precipitation (Li et al., 2021). At the same time, the most influential variables for water yield were precipitation and evapo-transpiration, since they are the determining factors in the variations of water production in productive land uses.

#### 3.1.3 The water regulation index

**Figure 5:**
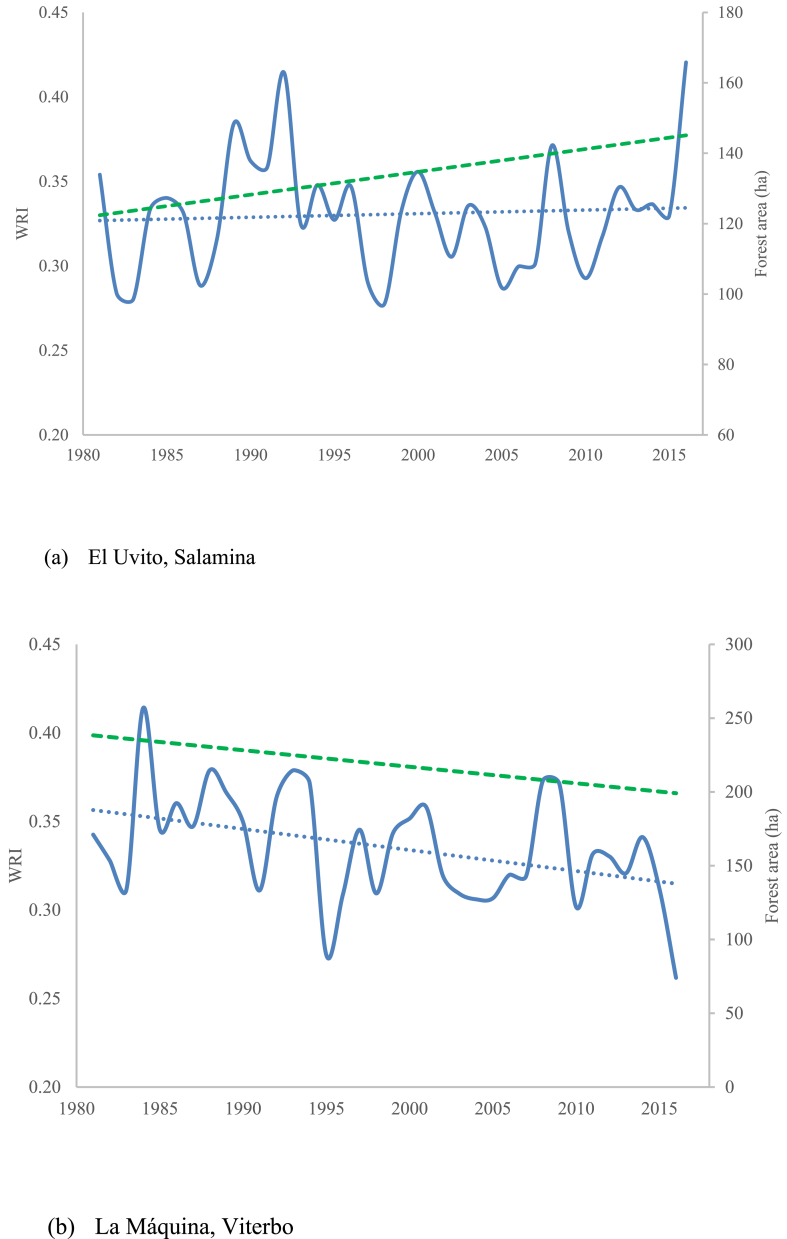
Historical behavior of RHI and forest cover. Doted green line is forest cover, continuous blue line is WRI, and dotted blue line is a WRI trend line.

The results for the WRI (Equation 1) of the two cases were for El Uvito (Salamina municipality) = 0.20, and for La Máquina (Viterbo municipality) = 0.23, which represents low levels of hydrological regulation according to table (Table 1). In addition, we found that while the WRI in El Uvito has been constant, and forest cover has been increasing throughout 35 years, La Máquina shows a decreasing pattern both in WRI and forest cover during the same period (Figure 5).

Regarding the scenario of 100% forest area, we found that the WRI of El Uvito increases to 0.39 and La Máquina to 0.50; on the other hand, the results were 0.17 for El Uvito and 0.008 for La Máquina only with one ha of forest cover.

#### 3.1.4 The matrix

To develop the matrix, we invited 10 people from different institutions, such as Corpocaldas, VivoCuenca (NGO), University of Manizales, University of Caldas, Catholic University of Manizales and independent consultants in the fields of biology and ecosystems research. After a written systematic description, they did this through online Excel matrices. Thus, according to the results presented in Table 3, the ES with the highest level of agreement in the rating was water provision (*α = 0.79*), followed by hydrologic regulation (*α =0.76*).

**Table 3:** Ecosystem capacity to provide ES.

El Uvito (Salamina)			Regulating	*α = 0.79*	Material	*α =0.76*
Ecosystem/land cover	Area (ha)	Land cover area (%)	Water regulation ranking (1–5)	Adjusted∗ ranking	Water supply ranking (1–5)	Adjusted ranking
Forests	147.9	26%	5	1.31	4.4	1.2
Pastures	255.69	45%	1.1	0.50	0.9	0.4
Heterogeneous agricultural areas	160.37	28%	2.8	0.28	2.2	0.6
	563.96	100%	2.97	2.09	2.50	2.20
La Máquina (Viterbo)		Regulating	*α = 0.79*	Material	*α =0.76*	
Ecosystem/land cover (Ha)	Area (ha)	Land cover area (%)	Water regulation ranking	Adjusted∗ value	Water supply ranking	Adjusted value
Forests	15.81	4%	5	0.20	4.4	0.89
Pastures	9.31	2%	1.1	0.03	0.9	0.02
Heterogeneous agricultural areas	367.54	94%	2.8	2.62	2.2	2.06
	392.66	100%	2.97	2.85	2.50	2.97

∗ The adjusted ranking refers to the fact that the rankings provided by the experts were adapted to the percentage of the specific land cover with respect to the total area. For example, in El Uvito, the forest has a rank of five for water regulation; however, not all of the area is covered by forest, so this value of 5 only explains 26% of the total capacity of the area to regulate water.

According to the table, the forest is an ecosystem which a very high capacity to provide water ES. On the other hand, the areas with herbaceous vegetation also have a relevant capacity, and pastures highlighted their low capacity. However, when analyzing the mixed ES generation capacity from the different covers, we found that for water regulation and water provision the values were middle-low. For instance, in Salamina, water regulation *= 2.09*, and water supply *= 2.2*, and for Viterbo, water regulation *= 2.85*, and water supply *= 2.97*.

#### 3.1.5 Social perceptions and the monetary value

We applied in total 507 questionnaires (Salamina = 318, Viterbo = 189). To see the questionnaire and the socio-demographic data related to the questionnaire, see Supplementary material A2, Table S2. What we want to highlight is that most people in both municipalities live in the area more than 30 years ago (Salamina = 36 years, Viterbo = 37 years), and most people in Viterbo Village know the supply area La Máquina (78% said yes), while in Salamina only 20%. When asking about the perceived contribution of both areas for water conservation, respondents declared mostly high levels (Salamina El Uvito = 4.36, Viterbo La Máquina = 4.25). However, respondents manifested current low levels of conservation of them (Salamina: 3.13, Viterbo: 3.39). In addition, Table 4 depicts rankings assigned to ES related to water in the supply areas where we found difference between the rankings assigned in both areas.

**Table 4:** Rankings assigned to water ES.

	El Uvito (Salamina)	S.E	La Máquina (Viterbo)	S.E
	average		average	
Water ES∗	4.94∗∗	.01	4.79	.04

∗ Water is considered as water regulating and water supply, given the cognitive burden.

∗∗ Mann-Whitney U-test *p < 0.01*.

Regarding the WTP analysis, we performed a mixed-effect logistic regression in order to identify the determinant attributes of the payment in each supply area. All the attributes were significant with *p= 0.00* and *p* < 0.05 (Supplementary material A2, Table S2). After running the regression, we calculated the marginal values associated with attributes assigned in the choice experiments. In the context of Salamina, the highest marginal value relates to maintaining water regulation (COP$ 12,377.50), while in Viterbo was to Maintain Forest cover (COP$ 29,200.00). Now, considering the aggregated values in both municipalities (total population), we found that the total monetary value per year identified in Salamina was COP$ 1,862, 774, 976 (USD$ 467,798.84), and for Viterbo was COP$ 894, 076, 236 (USD$ 224,529.44). Values of the supply areas per hectare per year were EL Uvito = USD$ 829, and La Máquina = USD$ 572.

### 3.2 The normalization

Once the values were recognized, we proceeded to normalize them between zero and one in order to make a general understanding of the values in the supply areas (Table 5). Here we found that water ES had higher normalized values since the Social dimension. For both supplying areas, *S* > *E*_*1*_*, E*_*2*_*, E*_*3*_, *E*_*4*_ and *S*_*1*_*> M*. In addition, we highlight that *E*_*1*_*, E*_*2*_*, E*_*3*_*, E*_*4*_>*M*. On the other hand, we found that, in general, El Uvito yielded higher values than La Máquina, except for *E*_*4*_ and *E*_*2*_. In both areas, people considered them as very important for water conservation (*S > 0.8*). However, it is important to stress that these social perceptions differed from the actual capacity of the supply areas to regulate water. For instance, the water regulation index in the area showed that *E*_*4Uvito*_ *= 0.20*&*E*_*4Máquina*_ *= 0.23*, a very contrasting value with *S*_*Uvito&Máquina*_ *> 0.80*; therefore, *S > E*_*4*_. The same happened with the ES capacity matrix results (*E*_*1*_*& E*_*2*_*< S*_*Uvito&Máquina*_ *> 0.80*). Finally, InVEST results for water yield (*E*_*3Uvito*_ *=0.42 & E*_*3Máquina*_ *= 0.39 < S*_*Uvito&Máquina*_*> 0.80*) depicted that the real biophysical capacity of the supply areas is below the social perceptions. Finally, considering the results, economic values in both areas were marginal (*M*_*Uvito*_ *= 0.05 & M*_*Máquina*_ *= 0.03*), which represents that value expressed in monetary terms should be the nuanced and very context-specific criteria for managing both supply areas.

**Table 5:** Normalized values of water Ecosystem Services.

Valuation layers	Source	Value types	Scale (*min- max*)	Units	El Uvito		La Máquina	
					Value	Normalized	Value	Normalized
Water conservation (capacity)	PreB survey	Social –***S***	1–5	Likert scale	4.36	0.84	4.25	0.81
Water regulation	ES-C matrix	Ecological -***E***_***1***_	1–5	Likert scale	2.09	0.27	2.85	0.46
Water provision	ES-C matrix	Ecological -***E***_***2***_	1–5	Likert scale	2.20	0.30	2.97	0.49
Water yield	InVEST	Ecological -***E***_***3***_	209- 2,288	Millimeters	1,083	0.42	1,030	0.39
Water regulation index	WRI index	Ecological -***E***_***4***_	0–1	No units	0.20	0.20	0.23	0.23
Monetary value	PreB survey	Monetary -***M***	0- 48,311∗	Int$/hectare/year	2,441∗∗	0.05	1,683∗∗	0.03

∗ Sum of the values of water provision (47,869 Int$/hectare/year) and regulation of water flows (442 Int$/hectare/year). Values are reported in de Groot et al. (2020) for the 2020 prices level. ∗∗ Value adjusted to Int$/hectare/year.

### 3.3 The articulation

El Uvito is a supplying area that is considered as being the future water source of Salamina's urban area. To do so, the Environmental Authority promotes land use conditioning in which the main use relates to conservation and restoration. People considered that El Uvito can support water conservation (*S = 0.84*), which was highlighted too in the focus groups. However, the current environmental state in the area demonstrates that the supplying area in fact has a low capacity to regulate water (*E*_*4*_ *= 0.20),* no matter the higher values given by experts' judgment on its capacity (*E*_*1*_ *= 0.27* & *E*_*2*_*= 0.30*). This calls attention to the need to carefully manage both the land use/cover and the dependence on water in the area. We say that, because considering the 100% forest-cover scenario in the water regulation index, the value does not grow significantly (*E*_*4*_ *= 0.20 < E*_*4100%forest*_*= 0.39*), which imposes an ecological limit for water use in Salamina. In addition, both community and decision-makers’ expectations in the area should be nuanced, given the mismatch between the real vs the expected capacity for water conservation/provision. Therefore, following our previous strategy of involving the environmental authority in the research process, after discussion we conjointly recommend –considering the plural values here identified the next guidelines:

1. Promote, as much as possible, land use/covers, which improve water regulation (in line with environmental authority's land-use guidelines).
2. Resize the water dependency of the ABACO; rethink the true water supply for the city, in the future.
3. Provide a more objective expectation for local people regarding the capacity of the area to provide water, in order to prevent conflicts given social and ecological mismatches.
4. Inform to the involved participants of the real capacity of the ABACO to provide water ESs.

The scenario is similar in La Máquina, but considering some particular aspects: first, La Máquina is a current source of water to the municipality (Viterbo), and second, the hydrological capacity with a 100% forest cover grows in a more important fashion (*E*_*4*_ *= 0.23 < E*_*4100%forest*_ *= 0.50*). Then, the recommendations are the same but nuanced. The third aspect to stress is that, considering the border conflict present in this supplying area, besides making visible the aforementioned recommendations, some additional guidelines should be considered:

1. Visualize the trade-offs between municipalities to face the shared conflict.
2. Promote a short and middle term strategy to prevent external conflicts, by changing *in situ* management activities in the “offender municipality”, and promoting in situ mitigation activities in the affected area.
3. As a final guideline, a compensation process could be promoted in order to manage external land-use conflicts. This compensation could consider the value obtained of USD$ 572 ha/year for management in La Máquina. However, this should not be considered the main strategy, given that *S > E > M.*

Finally, as a general guideline is the possibility to promote a land use related to nature-based tourism, given that in both supply areas this strategy was highlighted. Although this is a conditioned land-use for them, we highlight that social valuation regarding the area were high, which could be an additional argument to support this kind of activities in the areas.

## 4 Discussion

### 4.1 Methodological contributions

With our results, we add arguments regarding the recognition that the application of one single method and value type affects the outcomes of valuation (Jacobs et al., 2016). As we found, each method we applied depicted a particular valuation outcome (Table 5), and when we articulated them, we were able to understand the valuation scenario beyond a monistic approach. Therefore, we accept our first assumption where *the same ecosystem service is valued differently taking into account different approaches and scales,* but also, we stress that and *the results of a plural valuation comprehensively inform the decision-making process*.

We consider that PV issue of scale and value types, and we highlight Dendoncker et al. (2018) conclusion on depending the scale of the study area, different ES might be assessed, and hence different values captured. Likewise, PV let us identify additional values to society for addressing suitable strategies for enhancing sustainability (Peh et al., 2016). For this reason, we point out that our approach was useful to include different values, including monetary units under some particular conditions and purposes (Kallis et al., 2013), and highlighting different value languages encompassing ecological, social and monetary. This is what Himes et al. (2020) stress, about the need to include the value dimension and the different languages associated with ES by the involved participants at different scales.

Our results also point out that the plurality of values might not be necessarily reflected in standardized frameworks (Ebner et al., 2022). It means, that following the recommendation of mainstreaming PV, we stress that, what should be mainstreamed is the worldview instead of the values or methods for decision-making. Although we used two study-cases to apply our multi-layered approach, and we found some similarities in the valuation outcomes, the strategies we proposed were nuanced according to each area (see the different guidelines). If we see the big picture, we found in both supplying areas that *S > E > M*; however, what represent *S*, *E* and *M* in each supplying area differs. For instance, values related to water were different (Table 4), monetary values and *status quos* were contrasting, and eves ecological values differed according to changing scenarios (100%forest, 1 ha forest). Moreover, the areas had differentiated problems and pressures (see focus groups results). Then, as values are context-specific, our multilayered valuation attested to be an accurate strategy.

On the other hand, applying our design with a sequential mixed approach proved to be valuable. Some authors have reinforced that qualitative –constructivist or quantitative approaches based on social perceptions are adequate for a PV (Ebner et al., 2022; Gale and Ednie, 2020; Tusznio et al., 2020; Arias-Arévalo et al., 2017). What we can say with our results is that it is necessary to highlight ecological values (beyond social constructions or ratings), because if we had based our analysis on the community perspective, we would have lost the identification of ecological constraints for ecosystem use (*S > E*). In other words, applying a mono-method approach could be insufficient and problematic (e.g. only social perceptions without ecological support, or only WTP without ecological support). For this reason, we found valuable the results provided by InVEST and the water regulation index, which contrasted with expert-based consultation and community perceptions. This represents a normal aspect in ES valuation, which is juxtaposition of values (Manzolli et al., 2022; Peck and Khirfan, 2021; Suarez et al., 2021). We are aware on the complexity of applying several methodological steps, however, PV studies should face this load because resources, time and skills are necessary (Maydana et al., 2020; Lopes and Videira, 2019).

### 4.2 Philosophical perspective

We highlight that we framed our multi-layered valuation in a Pragmatic philosophy of science. By one hand, this approach let us to understand manifested consequences in both areas rather than antecedent conditions; on the other hand, our results concerned with applications and problem solutions (Creswell and Creswell, 2018; Melnikovas, 2018), as we depicted through our approach. This goes in line with the initial aim of this research and the way in which we formulated and performed it. In this sense, we understand our results since a plural but reactive process of valuation aimed to short-term management in both supplying areas. Although we provided valuable results, we want to stress some philosophical constrains mainly since an ontological perspective.

Jacobs et al. (2016) contributing on the basis of the new valuation school, call for avoiding ontological/epistemological debates on how a context should be framed or “reality” analyzed, by focusing on practical outcomes using multiple methods. However, given that a pragmatist worldview does not address reality or structural factors that shape reality (Elder-Vass, 2022), with this perspective we just addressed the context in both supply areas in a multi-layered fashion but only empirically. A more comprehensive paradigm such as Critical Realism (Bhaskar et al., 2010; Bhaskar, 2008) depict a stratified ontology where the empirical is only a first layer of reality shaped by events and structures, and then, it should be considered for further research with structural and long-term implications in the interface ES valuation-management (Spash, 2012). In addition, it will allow better process of theorization and comprehension of the phenomena under study.

## 5 Conclusions

Firstly, what we want to point out with this paper that there is not a plural value of ES in a particular context; rather, there are plural values. It means, plural values of ES are a subset of social values, ecological values, and monetary values, which interact in specific contexts. In addition, we stress that the more layers assessed, the more values would appear in valuation scenarios. These values could be coherent each other or could be in conflict, reason why is core to recognize them.

Our multi-layered valuation was useful in identifying conflicting perspectives and allowed us to understand the valuation context in both case studies. We emphasize that this is a demanding process in terms of time, resources and skills, but it is a necessary step to address the issue of PV. In fact, a more comprehensive valuation should be conducted by including not only a single ES, but also several ES. This will be useful to mainstream PV studies. On the other hand, our multi-method design demonstrated to be accurate not only to obtain social perceptions, but also ecological metrics and monetary units. Likewise, with this approach we contributed to highlight that there are ecological limits that should not be trespassed in order to achieve sustainability.

Furthermore, we agree with and contribute to the substantial body of literature, which states that monetary values do not represent the overall value of ecosystems; in fact, in the cases of our study it was the lowest value. Therefore, decisions should more predominantly consider ecological and social values, and consider monetary metrics only in very specific cases. Moreover, we conclude that there are social perspectives that do not coincide with ecological realities. This should be addressed to prevent future conflicts. Finally, the normalization process did not serve to simplify values, but to put them in context.

Finally, we consider valuable the joint participation of academics and the environmental authority professionals in shaping our research process. It was important not only at focusing on specific areas of study, but also at identifying specific problematic content and consolidating the results from a decision-making perspective.

## Declarations

### Author contribution statement

A. Suarez: Conceived and designed the experiments; Analyzed and interpreted the data, Contributed reagents, materials, analysis tools or data; Wrote the paper.

C.A Ruiz-Agudelo: Conceived and designed the experiments; Wrote the paper.

P. Arias-Arevalo: Conceived and designed the experiments; Analyzed and interpreted the data; Wrote the paper.

G. Flórez-Yepes; N. Arciniegas; L.A Vargas-Marín; A. Marulanda; E. Castro-Escobar Vargas-Marin; D. Blanco: Performed the experiments; Wrote the paper.

J. Ramirez; J.C Bastidas: Analyzed and interpreted the data; Wrote the paper.

### Funding statement

This work was supported by Corpocaldas and Universidad de Manizales (177-2021).

### Data availability statement

Data will be made available on request.

### Declaration of interest's statement

The authors declare no conflict of interest.

### Additional information

No additional information is available for this paper.

## Appendix A Supplementary data

Supplementary_Materials_A1_A2_A3 and B_V2.docx Supplementary_Materials_A1_A2_A3 and B_V2.docx
