# Supplementary material for: Recognizing, normalizing and articulating: An approach to highlight plural values of water ecosystem services in Colombia
Source: Heliyon. 2022 Sep 17;8(9):e10622. doi: 10.1016/j.heliyon.2022.e10622 (PMC9508513; doi:10.1016/j.heliyon.2022.e10622)
Supplement: Supplementary_Materials_A1_A2_A3 and B_V2.docx [file mmc1.docx]

**Supplementary material A1**

Table S1. Sources of information InVEST

| Data Type | Variable | Source/reference |
| --- | --- | --- |
| Climatic | Reference Evotranspiration | Global PET – Annual. CGIAR, 2019. |
|  | Precipitation | Historical Precipitation. Wordclim, 2017. |
| Biophysical | Land Use | National Land cover map. IDEAM, 2018. |
|  | Depth to root restricting | Soil Maps of the Colombian Territory. IGAC, 1998-2017. |
|  | PAWC | SoilGrids. ISRIC, 2017. |
| Hydrographic units | Watersheds | National hidrographic zonification. IDEAM, 2013 |

**Supplementary material A2**

Table S2. Socio-economic characteristics

|  | Salamina (n=318) | | Viterbo (n= 189) | |
| --- | --- | --- | --- | --- |
| Variable | Freq. | % | Freq. | % |
| Gender |  |  |  |  |
| Male | 197 | 38 | 124 | 65 |
| Female | 121 | 62 | 55 | 35 |
| Live in |  |  |  |  |
| Urban area | 273 | 14 | 173 | 91 |
| Rural area | 45 | 86 | 16 | 9 |
| Employment |  |  |  |  |
| Yes | 65 | 20 | 140 | 74 |
| No | 253 | 80 | 49 | 26 |
| Incomes |  |  |  |  |
| 1 | 116 | 36 | 50 | 27 |
| 2 | 190 | 60 | 120 | 63 |
| 3 | 9 | 3 | 18 | 9 |
| 4 | 3 | 1 | 1 | 1 |
| School level |  |  |  |  |
| 1 | 26 | 8 | 18 | 10 |
| 2 | 113 | 36 | 71 | 38 |
| 3 | 123 | 38 | 77 | 40 |
| 4 | 45 | 14 | 20 | 11 |
| 5 | 10 | 3 | 3 | 1 |

Table S3. Mixed-effects logistic regression

|  | El Uvito | | | | | La Máquina | | | |
| --- | --- | --- | --- | --- | --- | --- | --- | --- | --- |
| Election | Coef. | SE | | z | P>z | Coef. | SE | z | P>z |
| Maintain Forest cover | 1.63079 | .28526 | | 5.72 | 0.000 | 2.0445 | .38349 | 5.33 | 0.000 |
| Increase Forest cover | 3.82729 | .47494 | | 8.06 | 0.000 | .55766 | .25785 | 2.16 | 0.031 |
| Maintain hydric regulation | 4.95105 | .93889 | | 5.27 | 0.000 | -1.0162 | .25570 | -3.97 | 0.000 |
| Decrease hydric regulation | 2.88155 | .57795 | | 4.99 | 0.000 | -1.8335 | .30099 | -6.09 | 0.000 |
| Increase productivity | -1.03046 | .29629 | | -3.48 | 0.001 | .92597 | .23927 | 3.87 | 0.000 |
| Maintain productivity | -1.37031 | .29672 | | -4.62 | 0.000 | .63973 | .30602 | 2.09 | 0.037 |
| Wtp | -.000403 | .00004 | | -9.66 | 0.000 | -.00007 | .00003 | -2.52 | 0.012 |
| _cons | -4.51477 | 1.0323 | | -4.37 | 0.000 | -1.5921 | .69575 | -2.29 | 0.022 |
| Alternative var(_cons) | 1.705189 | 1.44535 | |  |  | .990803 | .84196 |  |  |
| Obs.= 3,815 | | | | |  | Obs.= 2,280 | |  |  |
| Log likelihood = -1089.3523 | | | |  |  | Log likelihood = -854.57068 | | | |
| Wald chi2(7) = 160.43 | |  | |  |  | Wald chi2(7) = 71.11 | | | |
| Prob > chi2 = 0.0000 | | | | |  | Prob > chi2 = 0.0000 | | | |
| LR test vs. logistic model:  chibar2(01) = 119.79  Prob >= chibar2 = 0.0000 | | |  | | | LR test vs. logistic model: chibar2(01) = 71.44  Prob >= chibar2 = 0.0000 | | | |

Table S4. Marginal WTP

|  | El Uvito | La Máquina |
| --- | --- | --- |
| Level of the attribute | WTP*m* | WTP*m* |
| Maintain Forest cover | $ 4,076.98 | $29.200,00 |
| Increase Forest cover | $ 9,568.00 | $7.965,71 |
| Maintain hydric regulation | $ 12,377.50 | $-14.517,14 |
| Decrease hydric regulation | $ 7,203.75 | $-26.185,71 |
| Increase productivity | $ -2,576.00 | $13.227,14 |
| Maintain productivity | $ -3,425.75 | $9.139,00 |
| Total person/month | $ 27,224.48 | $18,829.00 |
| Aggregated year | $ 1,862,774,976 | $ 894,076,236 |
| $/Ha/year | $ 3,301,621.72 | $ 2,277,321.03 |

**Supplementary material A3**


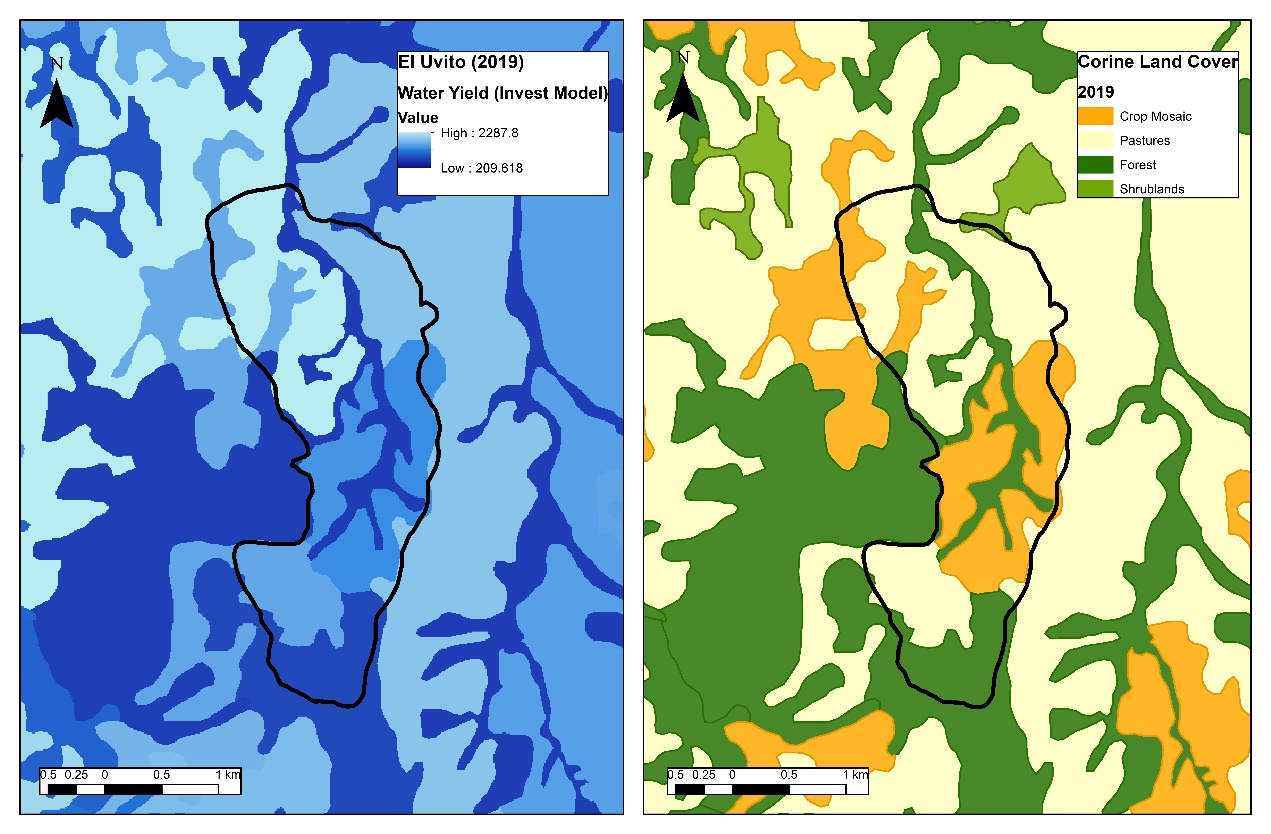


Figure S1. Supply area El Uvito


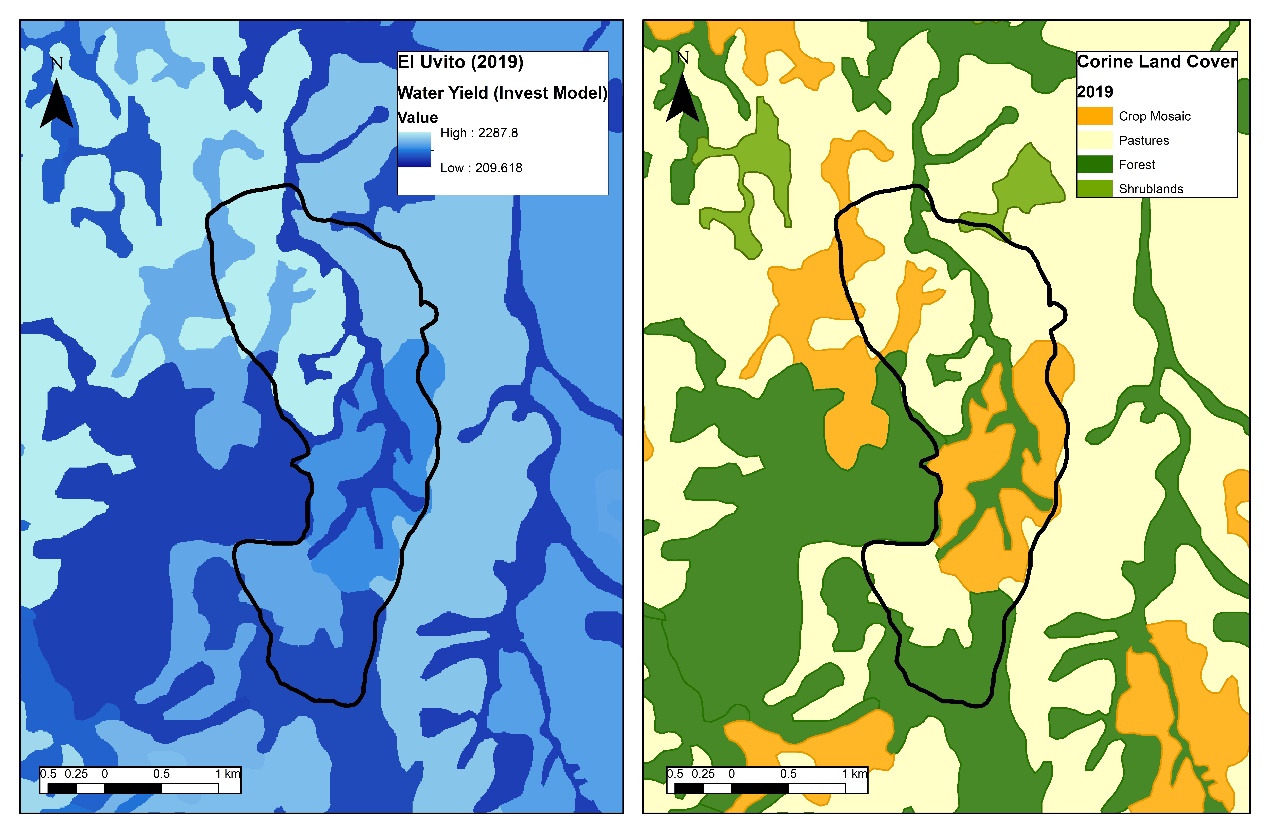


Figure S2. Supply area La Máquina

**Supplementary material B**

Questionnaire

Good morning/afternoon. My name is __________________________________. I would like to make a questionnaire in order to know your opinion about the state of the areas that supply water to the municipal aqueducts of the municipality. It will take you 5 to 10 minutes. The information you will provide is anonymous and absolutely confidential, your name will not appear in any case; the results of this research will be used to make a proposal to help the conservation of ecosystems.

- Do you agree to answer the questionnaire:

Yes: ____ No: ____

This work is related to the Water Supply Areas for Human Consumption or Micro-watersheds supplying aqueducts . This control point is defined by the catchment or intake site, located in a surface water supply source, of a water supply system for an urban or rural aqueduct.

**PART I: LOCAL PERCEPTIONS OF THE AREAS WHERE THE WATER IS TAKEN (Mention the AREA of the municipality of reference)**

1. Do you know the area of **El Uvito**):

Yes___ NO___

1. Rate how much you agree with the following statements (mark with an X in the appropriate box):

| **Afirmación** | Totally disaagree | Disagree | Indiferent | Agree | Totally agree |
| --- | --- | --- | --- | --- | --- |
| The area helps to conserve the municipality's water |  |  |  |  |  |

1. How conserved do you think the water supply area is?
2. Very bad conserved
3. Bad conserved.
4. Regular.
5. Weell conserved.
6. Very well conserved.
7. Please rate the following benefit provided by the area (mark with an X in the corresponding box):

| **Beneficio** | It does not benefit me at all | It does not benefit me | Indifferent | It benefits me | It benefits me a lot |
| --- | --- | --- | --- | --- | --- |
| Agua |  |  |  |  |  |

**PART II: SCENARIOS AND WILLINGNESS TO PAY**

Now I will present some alternatives about what you think of the area regarding environmental issues and productive activities.

"CORPOCALDAS wants to carry out a project to improve the environmental conditions of El Uvito, which is a water supply area. For this, it is necessary that the inhabitants of the municipality make a voluntary payment through the monthly water bill. Please note that you will have to choose the options that you like the most. If you choose scenarios with values to be paid monthly, please note that these will be through the water service bill". (Mark an X in the appropriate box)":

| 8&9 | **Alternative A** | **Alternative B** | **Alternative C** |
| --- | --- | --- | --- |
| Cover | Maintain | Maintain | Maintain |
| Water regulation | Decrease | Maintain | Maintain |
| Productive activities | Prohibits | Increase | Maintain |
| WTP | $ 10.000 | $ 3.000 | $ 0 |
|  | Choice (  ) | Choice (  ) | Choice (  ) |

| 7&4 | **Alternative A** | **Alternative B** | **Alternative C** |
| --- | --- | --- | --- |
| Cover | Decrease | Increase | Maintain |
| Water regulation | Improve | Decrease | Maintain |
| Productive activities | Increase | Increase | Maintain |
| WTP | $ 10.000 | $ 5.000 | $ 0 |
|  | Choice (  ) | Choice (  ) | Choice (  ) |

| 5&6 | **Alternative A** | **Alternative B** | **Alternative C** |
| --- | --- | --- | --- |
| Cover | Increase | Decrease | Maintain |
| Water regulation | Improve | Maintain | Maintain |
| Productive activities | Prohibits | Prohibits | Maintain |
| WTP | $ 3.000 | $ 5.000 | $ 0 |
|  | Choice (  ) | Choice (  ) | Choice (  ) |

| 3&2 | **Alternative A** | **Alternative B** | **Alternative C** |
| --- | --- | --- | --- |
| Cover | Decrease | Increase | Maintain |
| Water regulation | Decrease | Maintain | Maintain |
| Productive activities | Maintain | Maintain | Maintain |
| WTP | $ 3.000 | $ 10.000 | $ 0 |
|  | Choice (  ) | Choice (  ) | Choice (  ) |

**PART III: SOCIOECONOMIC INFORMATION**

1. Gender: M__ F__
2. Years living in the area: _____________
3. Age: ___________
4. Live in:
5. Rural
6. Urban
7. Working?
8. yes
9. No
10. Monthly incomes

| <1M | 1 |
| --- | --- |
| 1-2M | 2 |
| 2-3M | 3 |
| 3-4M | 4 |
| 4-5M | 5 |
| >5 | 6 |

1. Educational level

| None | 1 |
| --- | --- |
| Basic | 2 |
| High school | 3 |
| Tecnic | 4 |
| Profesional | 5 |
| Postgraduate | 6 |
